# Nucleophosmin1 and isocitrate dehydrogenase 1 and 2 as measurable residual disease markers in acute myeloid leukemia

**DOI:** 10.1371/journal.pone.0253386

**Published:** 2021-06-21

**Authors:** Petra Kövy, Zoltán Őrfi, András Bors, András Kozma, László Gopcsa, János Dolgos, Nóra Lovas, József Harasztdombi, Viktor Lakatos, Ágnes Király, Gábor Mikala, István Vályi-Nagy, Péter Reményi, Hajnalka Andrikovics

**Affiliations:** 1 Laboratory of Molecular Genetics, Central Hospital of Southern Pest National Institute of Hematology and Infectious Diseases, Budapest, Hungary; 2 School of PhD Studies Rácz Károly, Semmelweis University, Budapest, Hungary; 3 Department of Transfusiology, Semmelweis University, Budapest, Hungary; 4 Department of Hematology and Stem Cell Transplantation, Central Hospital of Southern Pest National Institute of Hematology and Infectious Diseases, Budapest, Hungary; The University of Adelaide, AUSTRALIA

## Abstract

Monitoring measurable residual disease (MRD) in acute myeloid leukemia (AML) plays an important role in predicting relapse and outcome. The applicability of the leukemia-initiating nucleophosmin1 (*NPM1)* gene mutations in MRD detection is well-established, while that of isocitrate dehydrogenase1/2 (*IDH1/2*) mutations are matter of debate. The aim of this study was to investigate the stability of *NPM1* and *IDH1*/*2* mutations at diagnosis and relapse retrospectively in 916 adult AML patients. The prognostic value of MRD was evaluated by droplet digital PCR on the DNA level in a selected subgroup of patients in remission. *NPM1* re-emerged at relapse in 91% (72/79), while *IDH1/2* in 87% (20/23) of mutation-positive cases at diagnosis. *NPM1* mutation did not develop at relapse, on the contrary novel *IDH1/2* mutations occurred in 3% (3/93) of previously mutation-negative cases. *NPM1* MRD-positivity after induction (n = 116) proved to be an independent, adverse risk factor (MRD^pos^ 24-month OS: 39.3±6.2% versus MRD^neg^: 58.5±7.5%, p = 0.029; HR: 2.16; 95%CI: 1.25–3.74, p = 0.006). In the favorable subgroup of mutated *NPM1* without *fms*-like tyrosine kinase 3 internal tandem duplication (*FLT3*-ITD) or with low allelic ratio, *NPM1* MRD provides a valuable prognostic biomarker (*NPM1* MRD^pos^ versus MRD^neg^ 24-month OS: 42.9±6.7% versus 66.7±8.6%; p = 0.01). *IDH1*/*2* MRD-positivity after induction (n = 62) was also associated with poor survival (MRD^pos^ 24-month OS: 41.3±9.2% versus MRD^neg^: 62.5±9.0%, p = 0.003; HR 2.81 95%CI 1.09–7.23, p = 0.032). While *NPM1* variant allele frequency decreased below 2.5% in remission in all patients, *IDH1/2* mutations (typically *IDH2* R140Q) persisted in 24% of cases. Our results support that *NPM1* MRD even at DNA level is a reliable prognostic factor, while *IDH1/2* mutations may represent pre-leukemic, founder or subclonal drivers.

## Introduction

Acute myeloid leukemia (AML) is an aggressive hematological malignancy with a rapidly evolving treatment paradigm. Although the majority of patients remain incurable, long-term remissions can be achieved in roughly one-third of these patients. The identification of prognostic markers bears outstanding relevance for optimizing treatment strategy. Measurable residual disease (MRD) after induction therapy and before hematopoietic stem cell transplantation is an independent, post-diagnosis prognostic indicator of relapse and survival. The application of molecular genetics and multiparametric flow cytometry are recommended for monitoring. Requirements for a reliable molecular genetic MRD marker are the following: (i) mutation burden fluctuates in parallel with leukemic tumor burden: present at disease onset, disappearing in remission and re-emerging at relapse, (ii) available method with the capability of achieving high sensitivity [1–3].

Nucleophosmin1 *(NPM1)* mutations are among the most frequently detected genetic alterations in AML (present in 25–35% of primary AML) defining a separate disease entity. *NPM1* frameshift mutations result in altered protein termination, loss of nuclear localization signals, and consequential abnormal cytoplasmic localization of the mutant protein [4–6]. Isocitrate dehydrogenase 1 and 2 (*IDH1* and *IDH2*) mutations occur in 7–14% and 8–19% of AML cases respectively. The gain-of-function mutations result in the production of an oncometabolite with consequential hypermethylation, gene expression alterations and impaired hematopoietic differentiation [4, 7].

*NPM1* alterations were reported as definite leukemia-founder mutations and optimal MRD markers. On the other hand contradictory data exist, whether *IDH1*and *IDH2* mutations represent pre-leukemic, or dominant clone mutations, therefore their value in MRD monitoring is not well established [3, 8]. In our study, we aimed to correlate *NPM1* and *IDH1* and *IDH2* mutational variant allele frequencies at diagnosis, remission and relapse to investigate the potential application of these mutations in MRD monitoring.

## Material and methods

### Patients

The study included 916 adult patients (449 males/467 females, median age at diagnosis 54 years; range: 16–94), consecutively diagnosed with AML between January 2001 and June 2020 in our Institute (Department of Hematology and Stem Cell Transplantation, Central Hospital of Southern Pest National Institute of Hematology and Infectious Diseases, Budapest, Hungary). In this patient cohort 253 patients were *NPM1*, 68 *IDH1* and 94 *IDH2* mutations positive (74 patients carried both *NPM1* and *IDH1/2* mutations). A significant proportion of patients 81% (n = 746/916) received curative treatment, out of which 26% (n = 176/746) was treated by allogeneic hematopoietic stem cell transplantation (HSCT). MRD monitoring was retrospectively evaluated in a selected subgroup of 116 *NPM1* (51 male/65 female, median age at diagnosis 48 years), and 62 *IDH1/2* positive patients (23 male/39 female, median age was 49 years). The inclusion criteria for the MRD monitored subgroup were the following: (i) curative chemotherapy; (ii) morphologic leukemia-free state (MLFS) after induction [1]; (iii) available DNA sample at diagnosis, after induction, and/or before HSCT. Patients with palliative therapy, death in aplasia or death from indeterminate cause, no MLFS after 2 courses of intensive induction treatment; unavailable DNA sample, or patients with rare undetectable *NPM1* or *IDH1/2* mutation were excluded from MRD evaluation. MRD was determined after induction and one month before HSCT if DNA samples were available. Data from AML patients diagnosed between 2001 and 2009 have already been reported in an earlier study [9] and *IDH1/2* data between 2001–2018 were presented in a Hungarian report [10]. Data collection was performed retrospectively. Definitions of *fms*-like tyrosine kinase 3 internal tandem duplication *(FLT3*-ITD) low and high allelic ratio, MLFS, overall survival (OS) and relapse-free survival (RFS) were described by European LeukemiaNet (ELN) 2017 recommendations [1]. The study was in accordance of the Declaration of Helsinki and was approved by the Institutional Review Board of Central Hospital of Southern Pest National Institute of Hematology and Infectious Diseases. Written informed consent was provided by all patients.

### Molecular genetic methods

Genomic DNA and RNA were isolated from bone marrow samples drawn at diagnosis, remission and relapse. Screening for hotspot mutations were performed from genomic DNA, at the time points of diagnosis and repeatedly at relapse by fragment analysis in case of *NPM1 (NPM1* diagnosis n = 916, relapse n = 161 if DNA was available); [11], and by high-resolution melting (HRM) or allele specific PCR in case of *IDH1/2* (diagnosis n = 842, relapse n = 116 if DNA was available) [9]. Positive cases were monitored with droplet digital PCR (ddPCR) after induction therapy (*NPM1* n = 116; *IDH1/2* n = 62; double positive = 33), before HSCT (1–30 days before; *NPM1* n = 38; *IDH1/2* n = 22), if DNA was available at that time point. Mutant *NPM1* RNA expression was also tested at diagnosis and after induction therapy (n = 39).

Diagnosis and follow-up samples of *NPM1* as well as *IDH1* and *IDH2* positive AML patients were investigated by ddPCR. For *NPM1* mutation detection primer and probe sequences are summarized in S1 Table [12–14]. *NPM1* type-A (c.860_863dupTCTG, p.Trp288CysfsTer12) specific reverse primer was described by Gorello *et al*. [13, 15]. A degenerate R primer (type-N) reported by Mencia-Trinchant *et al*. [14] was applied to detect *NPM1* mutations at the same position with different nucleotide insertions (c.860_863dupNNNN, p.Trp288CysfsTer12, referred as *NPM1* type-N mutation in this study). *GAPDH* was used as the reference gene for the assay for DNA [16], and *ABL1* for RNA [17]. Reactions were performed using Supermix for Probes (no dUTP) (BioRad), 900 nM of each primer, 250 nM of each probe, 100 ng DNA or 240 ng cDNA per well. For genomic DNA, assays were designed by Bio-Rad for the detection of the most common *IDH1/2* mutations (*IDH1* R132C ID: dHsaMDV2010053, R132H ID: dHsaMDV2010055 and *IDH2* R140Q ID: dHsaMDV2010057, R172K ID: dHsaMDV2010059). The PCR program started with an initial denaturation at 98°C for 10 min, 40 cycles of denaturation at 94°C for 30 sec, annealing at 55°C (for DNA) and at 60°C (for RNA) for 60 sec followed by enzyme deactivation at 98°C for 10 min. The QX200 Droplet Digital PCR System and QuantaSoft Software (Version 1.7.4.0917, BioRad) were used for the evaluation of the results.

The ddPCR measurements were acceptable if: (i) reference copies or total copies > 32,000, (ii) total droplet count >15,000; (iii) empty droplets > 100. MRD samples were measured in duplicate wells to achieve optimally more than 4.5-log sensitivity (at least 32,000 copies of reference gene) [18]. The ddPCR measurements were also performed in 20–35 mutation negative controls to determine the limit of blank (LoB = mean^negative samples^ + 1.645x standard deviation [SD]) and to determine the limit of detection (LoD = mean^negative samples^ + 3.3×SD) [19, 20]. Variant allele fraction (VAF) lower, than 2.5% correspond to <5% (pre)leukemic cells. Samples taken during MLFS (bone marrow blasts <5%; absence of blasts with Auer rods; absence of extramedullary disease) displaying >2.5% VAF were categorized as persisting preleukemic clones.

### Statistical analysis

Categorical variables were compared by the Fisher’s exact test, continuous variables by Mann-Whitney tests. Kaplan-Meier method with log-rank statistics were used to calculate OS and RFS [1]. After induction OS were calculated from the time point of diagnosis, RFS from remission irrespective from performing HSCT. Regarding pre-transplant MRD monitoring, comparisons of OS and RFS were performed from the time point of HSCT. Following univariate analysis, age, cytogenetics, *FLT3*-ITD allelic ratio [1], *NPM1*, white blood cell count (WBC) at diagnosis, and MRD status were included in a Cox proportional hazard model for OS and RFS. Hazard ratios (HR) and 95% confidence interval (95%CI) values were calculated. In order to identify the cut off discriminating between low and high MRD burden groups, HRs for OS were compared at six different limits (0.05%; 0.1%; 0.2%; 0.5%; 1% and 2%) for *NPM1* type-A and type-N separately and combined [21]. P values below 0.05 were considered as statistically significant. For the statistical analysis SPSS Statistics version 22 (Armonk, NY) was applied.

## Results

### Occurrence of *NPM1*, *IDH1/2* mutations in the total AML cohort

This study included 916 adult AML patients (S2 Table). Cytogenetic results were available for 94% (n = 861) of patients: favorable (n = 136; 16%), intermediate (n = 507; 59%) and adverse (n = 218; 25%) ELN 2017 cytogenetic risk categories were identified. *NPM1* mutation occurred in 28% (n = 253/916), *FLT3*-ITD in 25% (n = 226/916); *FLT3* tyrosine kinase domain mutations (*FLT3*-TKD) in 8% (n = 71/910); *IDH1* in 8% (n = 68/842) and *IDH2* in 11% (94/842). *IDH1* R132H associated with *NPM1* positivity more commonly than other *IDH1* R132 codon mutations: 90% (n = 27/30) versus 26% (n = 10/38), p<0.0001. Also *IDH2* R140Q co-occurred with *NPM1* in 49% (n = 37/76), while R172K never associated (p<0.0001).

**Fig 1:**
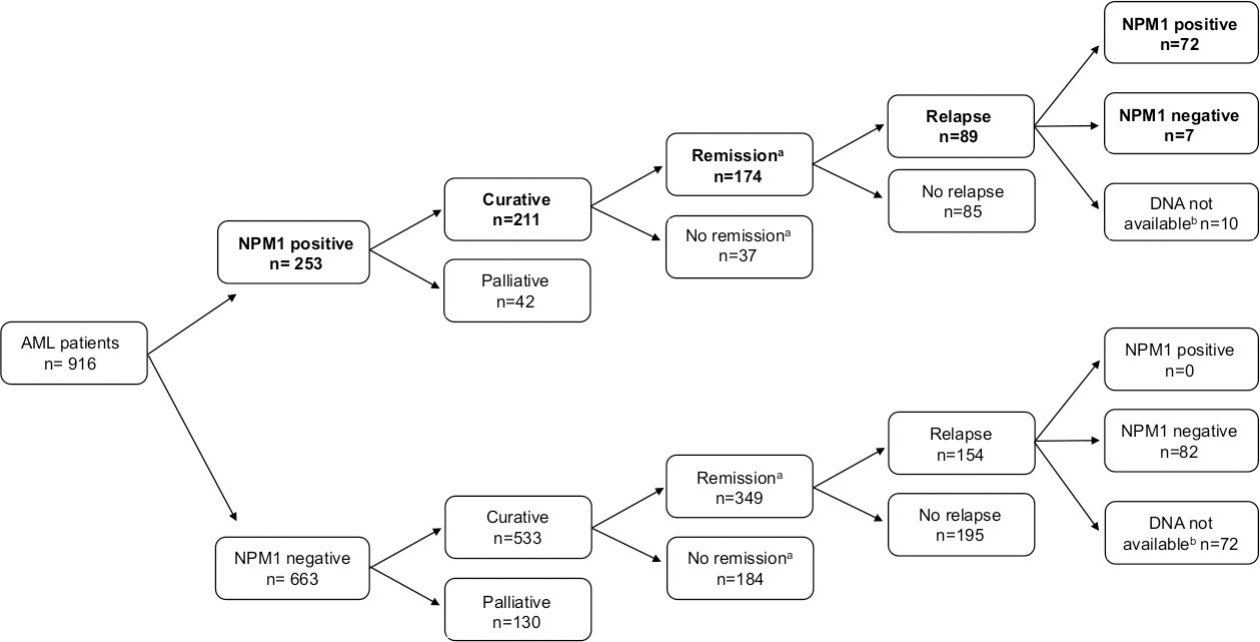
Clinical characteristics of *NPM1* positive AML patients. ^a^Remission was defined as morphologic leukemia-free state (MLFS) after induction. ^b^DNA not available at the time point of relapse.

In the *NPM1*-positive cohort, 211 patients were treated with curative intent, out of which remission (MLFS) was reached in 174 cases (Fig 1). The stability of *NPM1* mutation during disease evolution was studied with 79 paired *NPM1* mutant samples drawn at diagnosis and relapse. The *NPM1* mutation re-emerged at relapse in 91% of *NPM1* positive cases (n = 72/79). Time period from diagnosis till relapse was not significantly longer in cases where *NPM1* was undetectable at relapse compared to cases with persistent *NPM1* mutation at relapse [median 7.1 month (range: 0.1–172.2 month) versus 6.6 month (range: 2.2–152.9 month) respectively, p = 0.46]. All seven patients with clonal *NPM1* regression had normal karyotype at the time of diagnosis; one patient out of five with karyotyping available at relapse had clonal evolution (trisomy 8). None of our *NPM1* negative AML cases gained *NPM1* mutation positivity at relapse, among the 82 *NPM1* negative patients, where samples at diagnosis and relapse were available at both time points.

**Fig 2:**
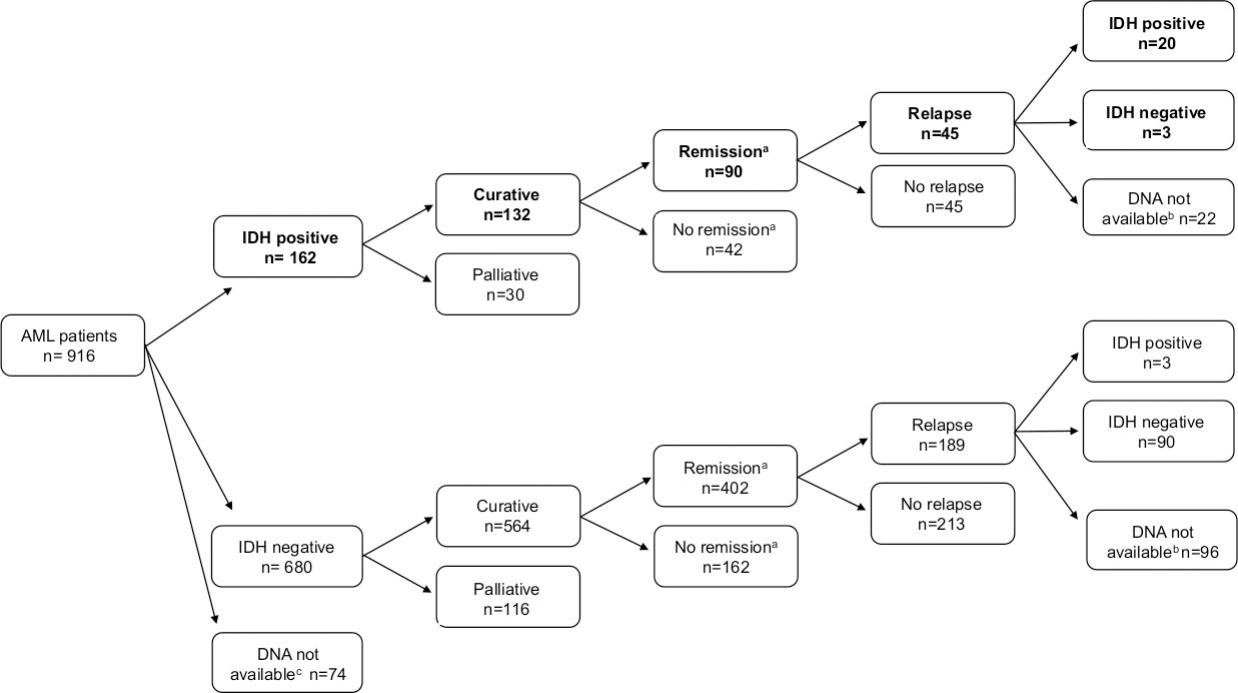
Clinical characteristics of *IDH1* and *IDH2* positive AML patients. ^a^Remission was defined as morphologic leukemia-free state (MLFS) after induction. ^b^DNA not available at relapse. ^c^DNA not available at diagnosis for *IDH1/2* testing.

In the *IDH1/2*-positive cohort (n = 162), 132 patients were treated with curative intent, out of which remission (MLFS) was reached in 90 cases (Fig 2). *IDH1/2* mutations were undetectable at relapse in 13% of the *IDH1/2*-positive cohort with available DNA (n = 3/23, 1 *IDH1* R132C, 1 *IDH2* R140Q and 1 *IDH2* R172K). Time from diagnosis till relapse was not proven to be significantly longer in cases where *IDH1/2* was undetectable at relapse compared to cases with persistent *IDH1/2* mutation at relapse [median 7.4 month (range: 2.2–11.4 month) versus 8.6 month (range: 0.83–57.2 month) respectively, p = 0.65]. Interestingly in three (*IDH1* R132H: n = 1; *IDH2* R140Q: n = 2) out of 93 *IDH1/2* negative AML cases where diagnosis and relapse samples were both available, *IDH1/2* mutations appeared only at relapse. These cases were re-evaluated by the more sensitive ddPCR method at diagnosis and VAF (0–0.23%) was under the detection limit of HRM and/or allele specific PCR in each case.

### The applicability of ddPCR methods

*NPM1*-positive patients screened by capillary electrophoresis at diagnosis were retrospectively typed with type-A and type-N primers using ddPCR. Out of 200 AML patients (53 samples were not available) 97% (n = 194) was proved to be *NPM1* type-A or type-N and not more than 3% (n = 6) could not be detected with type-N primer [14] (S3 Table). *IDH1* or *IDH2* mutations were screened by HRM and allele-specific PCR (S4 Table). Out of the 68 *IDH1* positive AML patients 39% (n = 27) was *IDH1* R132C, 46% (n = 30) *IDH1* R132H, 15% (n = 11) *IDH1* R132G/L/S/P. In case of *IDH2* positive AML, 81% (n = 76) harbored *IDH2* R140Q and 19% (n = 18) *IDH2* R172K. In our patient cohort, 93% (151/162) *IDH1/2* mutation positive patients carried an *IDH1/2* variant detectable with ddPCR. Interestingly, *IDH1* R132H was associated with *NPM1* type-A mutation in 48% (n = 13/27), while other *IDH1* R132 codon mutants and *IDH2* R140Q co-occurred with *NPM1* type-A mutation in 70% (n = 33/47; p = 0.08; S5 Table).

### *NPM1* MRD monitoring

The LoD for *NPM1* type-A ddPCR was lower than type-N ddPCR both in DNA and RNA settings (S6 Table). *NPM1* mutant VAF values in DNA and *NPM1* mutant expression levels in RNA were considered as MRD negative if below 0.01% (type-A) or below 0.05% (type-N). In case of *NPM1*, 174 *NPM1* positive cases reached MLFS after induction, MRD monitoring could not be performed in 5 cases due to technical limitations (*NPM1* mutation could not be detected by type-A or type-N primers), and in 53 cases due to non-available DNA. Basic characteristics such as gender, age at diagnosis, induction therapy, HSCT, and outcome (death in aplasia or in indeterminate cause, remission, relapse, cytogenetic and molecular genetic data) of *NPM1* positive and MRD monitored patients were included in S3 Table. In 116 *NPM1* MRD monitored patients, *NPM1* mutant VAF was reduced below 2.5% in all patients in MLFS after induction.

**Fig 3:**
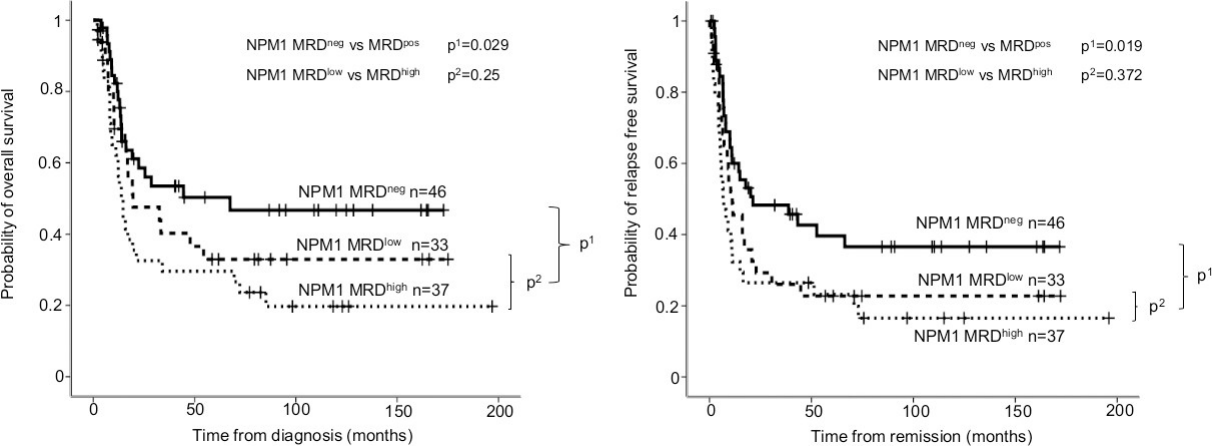
Probability of overall survival and relapse free survival according to *NPM1* MRD after induction. On both panels (A: overall survival; B: relapse free survival), the outcome of *NPM1* MRD-negative (MRD^neg^ VAF<0.01–0.05% depending on *NPM1* mutation type) and MRD-positive (MRD^pos^ VAF>0.01–0.05%) subgroups are shown with the associated p^1^ value. The *NPM1* MRD-positive subgroup was further divided in MRD low-positive (MRD^low^ VAF = 0.01–0.2%) and MRD high-positive (MRD^high^ VAF> 0.2%) subgroups, and compared with p^2^ values.

We examined the OS and RFS of 90 AML patients who have *NPM1* type-A mutation and further 26 patients who were monitored with type-N *NPM1* ddPCR from DNA. The median *NPM1* VAF at diagnosis was 45.7% (range: 11.5–49.3%), while after induction therapy was 0.06% (range: 0–2.5%). Out of the 90 patients with type-A *NPM1*, 35 patients were MRD-negative, and 55 MRD-positive. Favorable outcome measures were observed in MRD negative compared to MRD positive patients for *NPM1* type-A (24-month OS: 50.2±8.9% for negative versus 27.7±6.5% for positive, p = 0.010; and 24-month RFS: 40.2±8.6% versus 15.8±5.1% p = 0.009, S1 Fig). MRD-positive patients were further divided into MRD-low and MRD-high burden subgroups. In our patient cohort, *NPM1* VAF 0.2% was considered as the limit to discriminate between low and high burden: 28 patients were classified into the MRD low category (ranging from 0.01 to 0.2% *NPM1* mutant allele burden), and 27 patients in the MRD high category (above 0.2%). As expected even within the MRD positive subgroup, high allele burden cases showed a tendency to more adverse outcome measures (24-month OS: 40.6±10.3% for low versus 16.1±7.4% for high MRD, p = 0.088; and 24-month RFS: 19.4±7.8% versus 12±6.5%; p = 0.107, S1 Fig). Analyses were also performed for *NPM1* aggregate types-A and -N, and similar results were obtained, (24-month OS: 58.5±7.5% for negative versus 39.3±6.2% for positive, p = 0.029; and 24-month RFS: 48.3±7.5% versus 27.8±5.6%, p = 0.019, Fig 3). The difference did not reach the level of significance within the MRD positive group (24-month OS: 47.6±9.4% for low versus 32.6±8.0% for high, p = 0.250; and 24-month RFS: 29.3±8.2% versus 26.5±7.6%, p = 0.372, Fig 3). *NPM1* type-A and -N MRD positivity proved to be an independent risk factor in multivariate analysis beside age at diagnosis, cytogenetics and *FLT3-ITD* allele burden, and white blood cell (WBC) count above 100.000 per microliter at diagnosis (OS: HR 2.16 95%CI 1.25–3.74, p = 0.006; RFS: HR 2.21 95%CI 1.32–3.68, p = 0.002) (Table 1).

**Table 1:** Multivariate analysis of *NPM1* MRD status after induction.

	Overall Survival (n = 116)		Relapse Free Survival (n = 116)	
	Hazard ratio (95% CI)	P	Hazard ratio (95% CI)	P
***NPM1* MRD positivity**^**a**^	2.16 (1.25–3.74)	0.006	2.21 (1.32–3.68)	0.002
**Age (per year)**	1.02 (1.00–1.04)	0.019	1.02 (1.00–1.04)	0.053
**Cytogenetics**^**b**^	1.50 (0.86–2.63)	0.155	1.62 (0.94–2.82)	0.085
***FLT3*-ITD**^**c**^	1.75 (1.19–2.56)	0.004	1.74 (1.23–2.46)	0.002
**WBC >100.000/μL**	0.88 (0.50–1.56)	0.656	0.93 (0.54–1.58)	0.775

^a^*NPM1* MRD positivity was defined as VAF>0.01–0.05% depending on mutation type.

^b^Cytogenetics coded as normal karyotype (reference), other intermediate and adverse risk.

^c^*FLT3*-ITD coded in three categories as wild type (reference), low and high allelic ratio.

Abbreviations: 95%CI: 95% confidence interval; *FLT3*-ITD: *fms*-like tyrosine kinase 3 –internal tandem duplication; MRD: measurable residual disease; *NPM1*: nucleophosmin1; WBC: white blood cell count at diagnosis.

**Fig 4:**
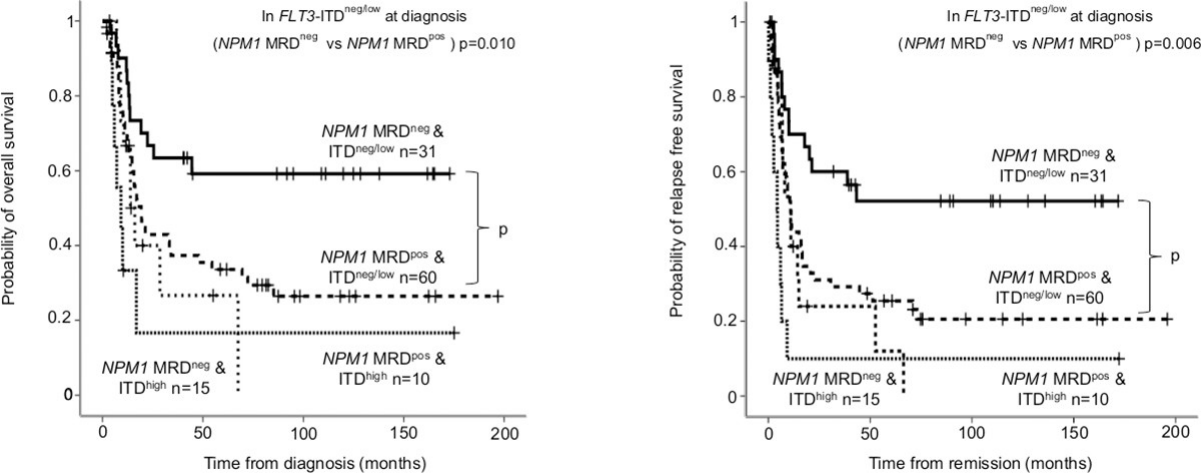
Overall survival and relapse free survival according to *NPM1* MRD stratified by *FLT3*-ITD allelic ratio. Based on the ELN 2017 genetic risk stratification, *NPM1* positive patients were categorized in to favorable (*FLT3*-ITD^neg/low^) and intermediate (*FLT3*-ITD^high^) subgroups. On both panels (A: overall survival; B: relapse free survival), further subgroups were established according to *NPM1* MRD after induction. *NPM1* MRD negativity was defined as VAF<0.01–0.05% depending on mutation type.

High allelic ratio of *FLT3*-ITD at diagnosis (categorized according to the ELN 2017 risk stratification) is a well-documented adverse risk factor in AML. In the favorable subgroup of mutated *NPM1* without *FLT3*-ITD (*FLT3*-ITD^neg^) or with low allelic ratio (*FLT3*-ITD^low^), the presence *NPM1* MRD provided a valuable prognostic biomarker (*NPM1* MRD^neg^ versus MRD^pos^ 24-month OS: 66.7±8.6% versus 42.9±6.7%, p = 0.010; RFS: 60±8.9% versus 31.1±6.2%, p = 0.006). *NPM1* MRD did not influence survival in the *FLT3*-ITD^high^ subgroup. *NPM1* MRD negative and *FLT3*-ITD high allele burden resulted similar survival measures to *NPM1* MRD positive patients (Fig 4).

**Fig 5:**
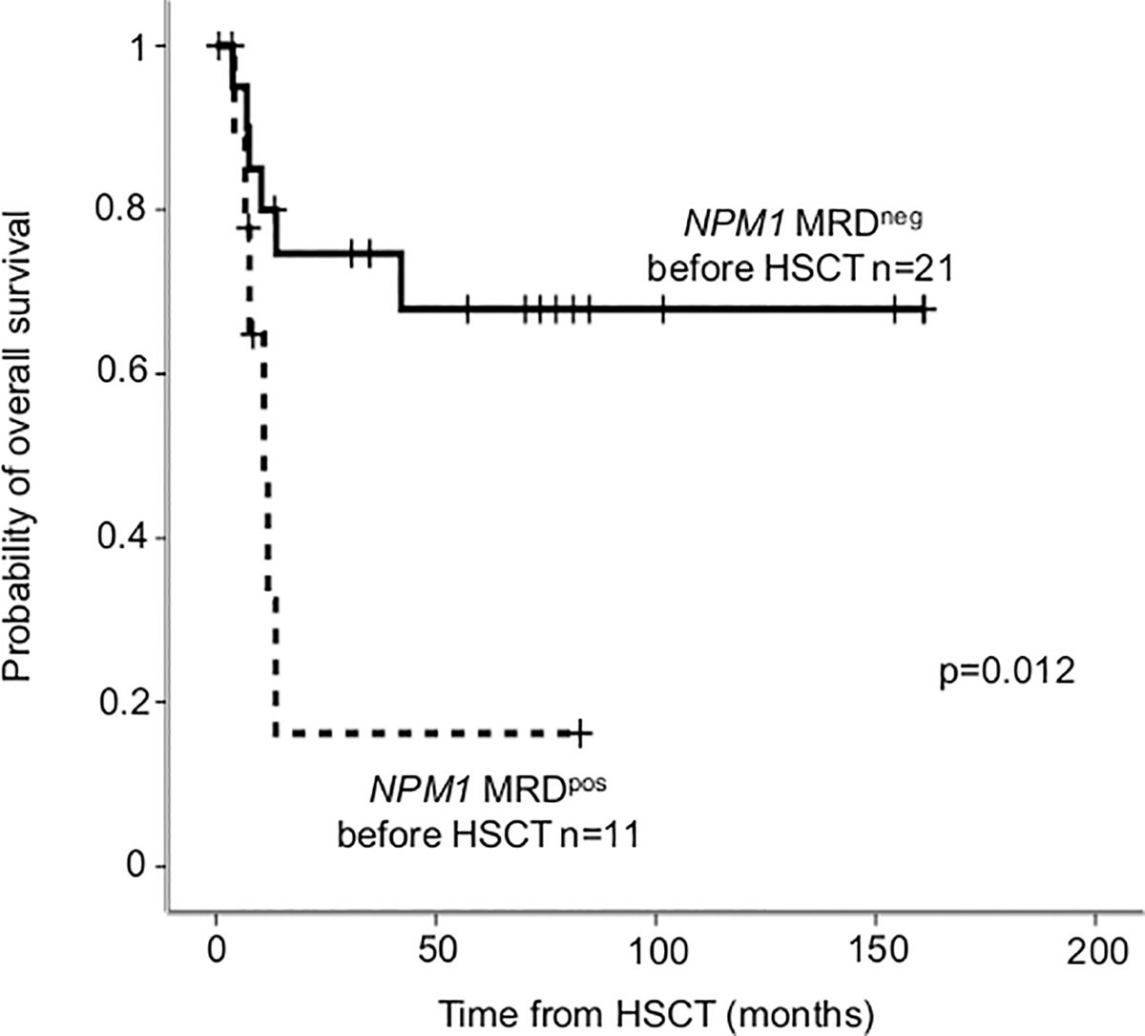
Overall survival according to *NPM1* MRD measured before HSCT. *NPM1* MRD negativity before HSCT was defined as VAF<0.01–0.05% depending on mutation type.

Out of the 38 patients who underwent allo-HSCT for MRD monitoring with the *NPM1* mutation type-A (n = 27) and type-N (n = 11), pre-HSCT sample was available in 32 (24 type-A and 8 type-N). *NPM1* MRD negativity before allo-HSCT proved to be favorable prognostic factor, OS after HSCT was significantly longer in MRD negative compared to positive patients (24-month OS MRD^neg^: 74.7± 9.8% versus MRD^pos^: 16.2±14.6%, p = 0.012; Fig 5).

Similarly to genomic DNA two or three log reduction was observable in mutant *NPM1* RNA expression (*NPM1*/*ABL1*, n = 39 patients) [at diagnosis: median 610.8% (range: 124.3–2882.4%), after induction: 1.0% (range: 0–398%)]. Despite the low number of RNA samples, high mutant *NPM1* expression after induction correlated with unfavorable outcome (24-month OS mutant *NPM1* expression <1%: 55.2±12.9% versus mutant *NPM1* expression >1% 20.0±11.9%, p = 0.005; and 24-month RFS: 51.6%±12.5% versus 12±7.9% respectively; p<0.001). We investigated parallel RNA and DNA-based *NPM1*^mut^ ddPCR methods from 39 samples after first induction therapy from 39 patient who have both DNA and RNA samples. The assay sensitivity proved to be higher on RNA samples. Altogether 46% of the RNA samples that displayed *NPM1*^mut^ expression (median: 0.1%; range: 0.01–5.1%) were detected as negative in the matching DNA samples (<0.01%). RNA assay (*NPM1*^mut^ expression) proved to be more sensitive (median: 1.3-log; range: 0.0–2.78-log) compared to DNA assay (*NPM1*^mut^ VAF) in samples with concomitant positivity both on RNA and DNA level (S2 Fig).

### *IDH1/2* MRD monitoring

The LoB for *IDH1/2* mutation detection was 0.06–0.08% and the LoD was 0.09–0.12% (S6 Table). In general, VAF below 0.2% for each *IDH1/2* form was considered as negative. In case of 90 *IDH1/2* positive patients in MLFS after induction, MRD monitoring could not be performed in 8 cases with *IDH1* R132G/L/S/P/ and in 20 cases with lacking DNA samples. Basic characteristics of *IDH1/2* positive and MRD monitored patients were included in S4 Table. We observed that *IDH1/2* VAF in morphologic leukemia free state was not reduced below 2.5% in 15 out of 62 cases (24%, 10 *IDH2* R140Q, 3 *IDH2* R172K, 1 *IDH1* R132H and 1 *IDH1* R132C). Seven cases were *NPM1* positive (6 *IDH2* R140Q and 1 *IDH1* R132H) at diagnosis but *NPM1* mutational burden was reduced below 2.5% in remission. Regarding the outcome of patients with persisting *IDH1/2* mutation: 9 patients relapsed and subsequently died, 2 patients alive after HSCT, 3 patients alive in complete remission after 12 months follow up and 1 patient died without relapse.

**Fig 6:**
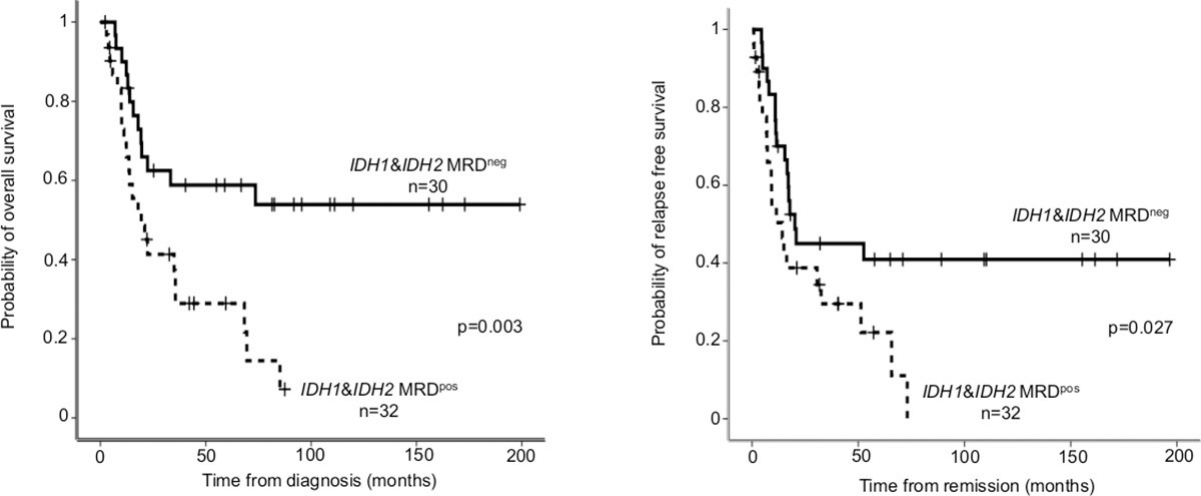
Overall survival and relapse free survival according to *IDH1/2* MRD after induction. On both panels (A: overall survival; B: relapse free survival) *IDH1/2* MRD negativity defined as VAF<0.2% and MRD positivity as VAF>0.2%.

In our analyses, the survival of *IDH1* or *IDH2* MRD-negative patients was significantly better than that of MRD-positive patients (24-month OS MRD^neg^: 62.5±9% versus MRD^pos^: 41.3±9.2%, p = 0.003; 24-month RFS: 45.0±9.3% versus 38.8±9.6% respectively, p = 0.027, Fig 6). In multivariate analysis, *IDH1/2* MRD positivity was proved to be an independent risk factor for survival besides age, cytogenetics, *FLT3*-ITD, *NPM1* and WBC (OS: HR: 2.81 95%CI: 1.09–7.23, p = 0.032, RFS: HR: 2.80 95%CI: 1.15–6.82, p = 0.023, Table 2).

**Table 2:** Multivariate analysis of *IDH1/2* MRD status after induction.

	Overall Survival (n = 62)		Relapse Free Survival (n = 62)	
	Hazard ratio (95% CI)	P	Hazard ratio (95% CI)	P
***IDH1/2* MRD positivity**^**a**^	2.81 (1.09–7.23)	0.032	2.80 (1.15–6.82)	0.023
**Age per year**	1.03 (0.99–1.06)	0.126	1.02 (0.99–1.05)	0.256
**Cytogenetics**^**b**^	1.98 (0.90–4.33)	0.089	2.36 (1.07–5.21)	0.034
***FLT3*-ITD**^**c**^	1.00 (0.42–2.38)	0.994	1.06 (0.49–2.28)	0.889
***NPM1***	1.62 (0.57–4.58)	0.364	2.26 (0.82–6.25)	0.115
**WBC >100.000/ul**	1.17 (0.49–2.78)	0.727	1.04 (0.45–2.43)	0.922

^a^*IDH1/2* MRD positivity was defined as VAF>0.2%.

^b^Cytogenetics coded as normal karyotype (reference), other intermediate and adverse risk.

^c^FLT3-ITD coded in three categories as wild type (reference), low and high allelic ratio.

Abbreviations: 95%CI: 95% confidence interval; *IDH1/2*: isocitrate dehydrogenase 1/2; *FLT3*-ITD: *fms*-like tyrosine kinase 3 –internal tandem duplication; MRD: measurable residual disease; *NPM1*: nucleophosmin1; WBC: white blood cell count at diagnosis.

**Fig 7:**
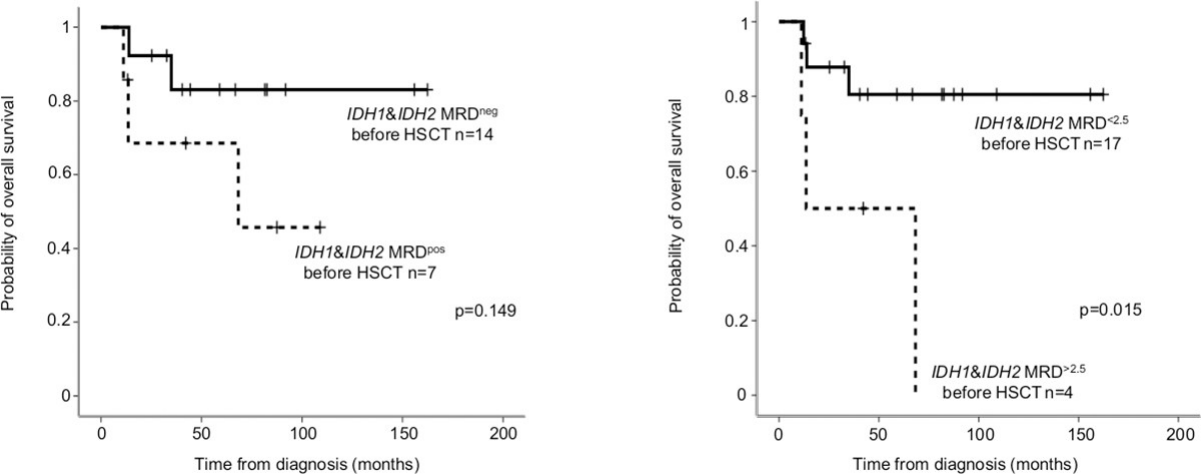
Probability of overall survival for *IDH1/2* MRD before HSCT. On panel A, *IDH1/2* MRD negativity before HSCT was defined as VAF<0.2%, on panel B as 2.5%.

In allo-HSCT cases, pre-HSCT samples were available in 21 out of 22 patients (10 *IDH1* and 11 *IDH2*). *IDH1/2* MRD negativity (VAF<0.2%) before allo-HSCT did not reach statistical significance (24-month OS MRD^neg^: 92.3±7.4% versus MRD^pos^ 68.6±18.6%, p = 0.149). *IDH1/2* MRD below 2.5% influenced significantly survival (24-month OS: MRD^<2.5%^ 87.8±8.1% versus MRD^>2.5%^ 50.0±25.0% before allo HSCT, p = 0.015; Fig 7).

## Discussion

The serial acquirement of somatic mutations in myeloid clone(s) was described as the multistep pathogenesis of AML. Several lines of evidence prove that *NPM1* mutations are responsible for the definitive acute leukemic transformation, therefore considered as leukemia founder mutations: (i) *NPM1* mutations are completely absent in the population without hematological malignancies even at a higher age [22–24]; (ii) *NPM1* mutations cannot be detected in AML patients months or years before the manifestation of AML [25, 26]; (iii) *NPM1* mutations occur rather rarely (approximately 2–3%) in preleukemic myeloid malignancies such as myelodysplastic syndrome (MDS) or in myelodysplastic/myeloproliferative neoplasm (MDS/MPN), which are mainly characterized by the progression to overt AML [27]; (iv) *NPM1* mutations were not present in preleukemic hematopoietic stem cells [28, 29]. Our observation that *NPM1* VAF decreased below 2.5% in all cases with morphologic leukemia-free state, also proved that *NPM1* mutations do not occur in the preleukemic state. (v) A further proof that *NPM1* mutations harbor leukemia-initiating properties is the high reappearance rate of *NPM1* in relapse, which was demonstrated as high as 86–100% in several clinical observations [30–39]. The long observational period in our study allowed us to detect late AML relapses. In line with previous studies less than 10% of our *NPM1* mutation positive cases relapsed as wild type *NPM1* AML. The loss of *NPM1* mutation in our patient cohort was not associated with longer remission before relapse, which was suggested by several previous studies [31, 32]. Although our study did not investigate the spectrum of preleukemic mutations, the persistence of *IDH2* R140Q mutation was observed in a single case with *NPM1*-mutation loss relapse 13 month after diagnosis.

Contradictory data exist in the literature, whether *IDH1* and *IDH2* mutations are preleukemic or AML founder mutations. Several studies suggest *IDH1/2* mutations as epigenetic modifiers as preleukemic events. (i) In large scale populational screening studies for clonal hematopoiesis of indeterminate potential (CHIP) mutations, *IDH2* R140 mutations were extreme rarely detected in elderly individuals (*IDH2* R140Q/W: 0.014%, four out of 29562 individuals) [22]. *(ii) IDH1* and *IDH2* mutations were detectable as premalignant, high-risk gene mutations years before the diagnosis of AML, but not in age-matched controls (8%; n = 15/188; three *IDH1* R132C/H/G and 12 *IDH2* R140 positive individuals with a median of 7 years before AML diagnosis) [26]. (iii) *IDH1* and *IDH2* mutations are also rarely present in preleukemic myeloid malignancies: 0.8–4% in chronic phase MPN, 4–14% in MDS, but its frequency increases up to 20–25% in blast phase transformation [40–43]. (iv) *IDH1* and *IDH2* mutations were detectable in preleukemic hematopoietic stem cells [28, 29]. The comparison of VAF values suggested that *IDH1* and *IDH2* mutations were more likely to develop before *NPM1* mutations [6]. The persistence of *IDH1*/*2* mutations (especially *IDH2* R140Q) in remission was observed in 7–39% of AML cases in the literature, [19, 44–46] which is in line with our study (24% of *IDH1* or *IDH2* mutations were detectable in complete remission with a higher than 2.5% VAF, 67% of persisting mutations was *IDH2* R140Q). The high mutational load in remission is a direct proof of preleukemic origin of the somatic mutation. This phenomenon in case *IDH1/2* mutations is not as frequent as in case of *DNMT3A*, *TET2*, *ASXL1* gene mutations, where reported rates vary between 51–82% [2, 47–49], (v) At relapse both *IDH1/2* gene mutations showed a relatively high stability (86–88% reported in publications, 87% in our study) similar that of *NPM1* mutation [31]. (vi) In *IDH1/2* mutation negative AML, the emergence of *IDH1* or *IDH2* mutations at relapse was observed in 10% in our study, which suggests the subclonal, late origin of these mutations. Interestingly, there is a usual mutation order in AML pathogenesis, but some mutations might appear both early and late events [31, 50].

A recent meta-analysis proved that lower MRD was consistently associated with improved outcome independently from applied method, sample source or sampling time of the assessment [51]. Regarding molecular genetic detection techniques, like quantitative PCR, digital PCR and next generation sequencing are extensively applied for MRD detection. High assay precision and reproducibility make ddPCR particularly suitable for MRD monitoring, which was reported in connection with several oncohematological drivers [52–56]. Individual assay designs make the quantification of multiple *NPM1* mutations challenging, but the application of degenerated primers allows the simultaneous detection of multiple *NPM1* mutations affecting the same localization (c.860_863dupNNNN) [14]. Our data and other studies also supported, that less than 5% of NPM1 mutations affects nucleotides at different positions [11, 38, 57].

Although consensus exists about the importance of *NPM1* MRD; broad range of heterogeneity was displayed concerning thresholds discriminating between low- and high-risk MRD. Studies comparing mutant *NPM1* transcript levels parallel in bone marrow (BM) and peripheral blood (PB) samples identified strong correlation, but an average of 1-log higher sensitivity in BM [21, 34, 38, 57–61]. In line with this observation, 3-log reduction of *NPM1*^mut^/*ABL1* transcript level was pointed as favorable prognostic indicator in BM, [21, 59, 60] but 4-log reduction was required in PB after induction therapy [57, 58]. In our study, bone marrow samples were processed. As *NPM1*^mut^ expression is highly abundant, greater sensitivity (median: 1.3 log, range: 0–2.78 log in our study) was achieved on RNA level than on DNA. *NPM1*^mut^ RNA expression level detection for MRD monitoring is recommended in the literature [13, 38]. Shayegi *et al*. investigated that 1% *NPM1*^mut^/*ABL1* expression corresponds to 0.016% *NPM1*^mut^ VAF or 1 in 32000 cells (1.8 log difference between RNA and DNA levels) [60]. These data suggested that *NPM1*^mut^ MRD screening should be performed on RNA expression, but in case of RNA unavailability, highly sensitive DNA methods can substitute. The applied cut-off for MRD negativity in our study (*NPM1* type-A: 0.01% and type-N: 0.05% on DNA level) corresponds approximately to 1% *NPM1*^mut^/*ABL1* expression level. We were unable to test large number of RNA samples, which is a major limitation of our retrospective study. Ivey *et al*. [57] demonstrated that RNA-MRD positivity in PB after induction (2 cycles) corresponded to higher cumulative incidence of relapse (MRC17 trial 3-year CIR: 82% versus 30%), similarly Balsat *et al*. [58] (ALFA-0702 trial: 2-year CIR: 55% versus 21%); Hubmann *et al*. [62] less than 3log-reduction in BM RNA-MRD (AMLCG 1999, 2004 and 2008 trial: 2-year CIR 77.8% versus 26.4%,); Kapp-Schwoerer *et al*. [34] less than 3-log_10_ BM or PB RNA-MRD (AMLSG 09–09 trial 4-year CIR BM: 60% versus 28.5%; PB: 62.5% versus 33.9%). On the DNA level, we also observed that MRD positivity (less than 3log reduction) was associated with adverse outcome, and DNA-MRD after induction therapy is capable to identify high-risk *NPM1*^mut^ patients.

The co-occurrence with *FLT3*-ITD was recognized as an adverse factor in *NPM1* mutant AML, due to the highly proliferative nature of the leukemic clone with ITD [38, 63]. Although *NPM1* mutation was referred as favorable or intermediate ELN prognostic categories depending on the presence of *FLT3*-ITD with high mutational load [1]. Recently, the reclassification of ELN prognostic criteria identified high *FLT3*-ITD load as adverse risk irrespective of *NPM1* mutation status [64]. Allogeneic HSCT in first complete remission is not recommended in favorable risk AML, on the other hand relapsed *NPM1*-positive cases have adverse outcome [65]. We observed that the measurement of *NPM1* MRD was capable to identify high risk patients even in the favorable risk *NPM1* positive AML without high ITD load. *NPM1* MRD negativity (*NPM1*^mut^ VAF<0.01–0.05% after induction) with high *FLT3*-ITD allele burden at diagnosis showed similarly adverse survival to *NPM1* MRD positive patients.

Molecular MRD measurements serve not only prognostic, but may influence therapy. In case of persistent MRD, HSCT consolidation improved survival over chemotherapy [66]. In ELN 2017 favorable risk *NPM1*^mut^ AML subgroup, molecular failure (defined as *NPM1*^mut^/*ABL1* >0.05% after consolidation or *NPM1*^mut^ reappearance after molecular response; which affected 40% of *NPM1*^mut^ cases) served as indication for allogeneic HSCT in first complete remission. MRD-guided approach involving early intervention resulted in improved outcome (two-year OS: 85% for HSCT-treated patients with molecular failure and 39% for patients with hematological relapse) [67]. For elderly or unfit patients, azacitidine was reported to prevent or delay hematological relapse in MRD-positive AML [68].

Our data investigating pre HSCT *NPM1*^mut^ MRD are in good concordance with other studies with similar MRD time-point assessment: pre HSCT MRD negativity predicts favorable outcome after HSCT [21, 66, 69–71]. Detection of MRD-positivity before HSCT guide therapeutic choices during conditioning and graft versus host disease prevention, e. g. preferably T-cell repleted versus T-cell depleted transplant [21]; preferably myeloablative versus reduced intensity conditioning [72]. MRD measurements can even guide targeted *FLT3*-inhibitor therapy identifying patients who benefit mostly [73].

The role of MRD-monitoring is well-documented in case of *NPM1*, but data are scarce about *IDH1* and *IDH2* mutations. We applied BioRad-designed mutation detection reagents on BioRad QX200 Droplet Digital PCR System, but interestingly we were not able to reach as high sensitivity as in case of *NPM1*. Similar technical limits (LoD: 0.2%) were reported in a previous study applying the same detection [19]. Our data also supported the preleukemic nature of *IDH1/2* mutations, but the persistence of *IDH1/2* mutations (VAF>2.5%) in complete remission was associated with adverse outcome, higher chance of relapse or the development of myelodysplasia [19, 44]. The presence of a preleukemic clone in morphologic leukemia-free remission was generally reported to associate with inferior survival compared to patients without persisting oncogenic mutations [74, 75]. On the other hand, persistent *DNMT3A*, *TET2*, *ASXL1* mutations were not connected with higher relapse rate and several reports described long-term remission even with high VAF [2, 47–49]. The frequency of persistent *IDH1/2* mutations in remission was reported as high as 7–39% depending on the VAF cut -off (1–5%) or on the applied chemotherapy [2, 19, 44, 45], which was similar to our observation (24%). In line with previous publications [19, 44, 45], our data also indicated that persisting *IDH1/2* mutations in remission were associated with adverse prognostic impact. Currently no guidelines exist whether pre-emptive therapeutic interventions (such as HSCT or *IDH1/2* inhibitors) could reduce relapse rate or improve survival in case of persisting *IDH1/2* mutations in remission. The combination of *IDH1* or *IDH2* inhibitors with intensive chemotherapy in newly diagnosed AML might improve mutation clearance, although no comparative data exist with or without the inhibitors [76].

In summary, we investigated a considerably large number of AML patients systematically over a long time, the limitation of our study is the retrospective study design and the heterogeneous treatment protocols applied during the observational period. Our results support that *NPM1* MRD even at DNA level is a reliable prognostic factor. On the other hand, *IDH1/2* mutations may represent pre-leukemic, founder or subclonal drivers, still *IDH1/2* MRD may also identify high risk AML. As MRD represents a biological continuum, special detailed guidelines are required to establish proper thresholds for the initiation of pre-emptive therapies.

## Supporting information

S1 Table Primers and probes used in *NPM1* ddPCR. (XLSX)

S2 Table Cytogenetic and molecular genetic characteristics of 916 AML patients. Abbreviations for S2–S5 Tables: DNR&AraC: standard daunorubicin&cytarabine regimen; *FLT3-ITD*: *fms*-like tyrosine kinase internal tandem duplication, *FLT3-TKD*: *fms*-like tyrosine kinase tyrosine kinase domain, HSCT: hematopoietic stem cell transplantation, *IDH*: isocitrate dehydrogenase, MLFS: morphologic leukemia-free state, MRD: measurable residual disease, *NPM1* mutation type not available*: patients with palliative treatment or with missing DNA samples were not further evaluated for *NPM1* mutation type.
(XLSX)

S3 Table Cytogenetic and molecular genetic characteristics of *NPM1* positive AML patients. (XLSX)

S4 Table Cytogenetic and molecular genetic characteristics of *IDH1* and *IDH2* positive AML patients. (XLSX)

S5 Table Baseline characteristics of *NPM1* and *IDH1/2* positive AML patients. (XLSX)

S6 Table Descriptives of the applied ddPCR methods. Abbreviations: LoB: limit of blank, LoD: limit of detection.
(XLSX)

S1 Fig Probability of overall survival and relapse free survival according to *NPM1* type-A MRD after induction. On both panels (A: overall survival; B: relapse free survival), the outcome of *NPM1* type-A MRD-negative (MRD^neg^ VAF<0.01%) and MRD-positive (MRD^pos^ VAF>0.01%) subgroups are shown with the associated p^1^ value. The *NPM1* type-A MRD-positive subgroup was further divided in MRD low-positive (MRD^low^ VAF = 0.01–0.2%) and MRD high-positive (MRD^high^ VAF> 0.2%) subgroups, and compared with p^2^ values.
(TIF)

S2 Fig Comparison of DNA and RNA based *NPM1* mutation MRD detection after induction. DNA based method describes the variant allele frequencies of mutant *NPM1* (*NMP1*^mut^/*GAPDH* ratio), while RNA method showed the *NPM1* RNA mutation expression (*NPM1*^mut^/*ABL1*). RNA samples that displayed *NPM1*^mut^ expression and VAF negativity in the are marked with the grey continuous lines (18 samples, 46%). Samples with at least 0.5 log higher RNA expression level with detectable mutant *NPM1* allele frequency on DNA level are shown with black dashed lines (19 samples, 49%). Only two samples (black continuous lines, 5%) showed equivalent *NPM1*^mut^ RNA expression and DNA allele burden.
(TIF)
