# Supplementary figures and images for: Nucleophosmin1 and isocitrate dehydrogenase 1 and 2 as measurable residual disease markers in acute myeloid leukemia
Source: PLoS One. 2021 Jun 21;16(6):e0253386. doi: 10.1371/journal.pone.0253386 (PMC8216517; doi:10.1371/journal.pone.0253386)

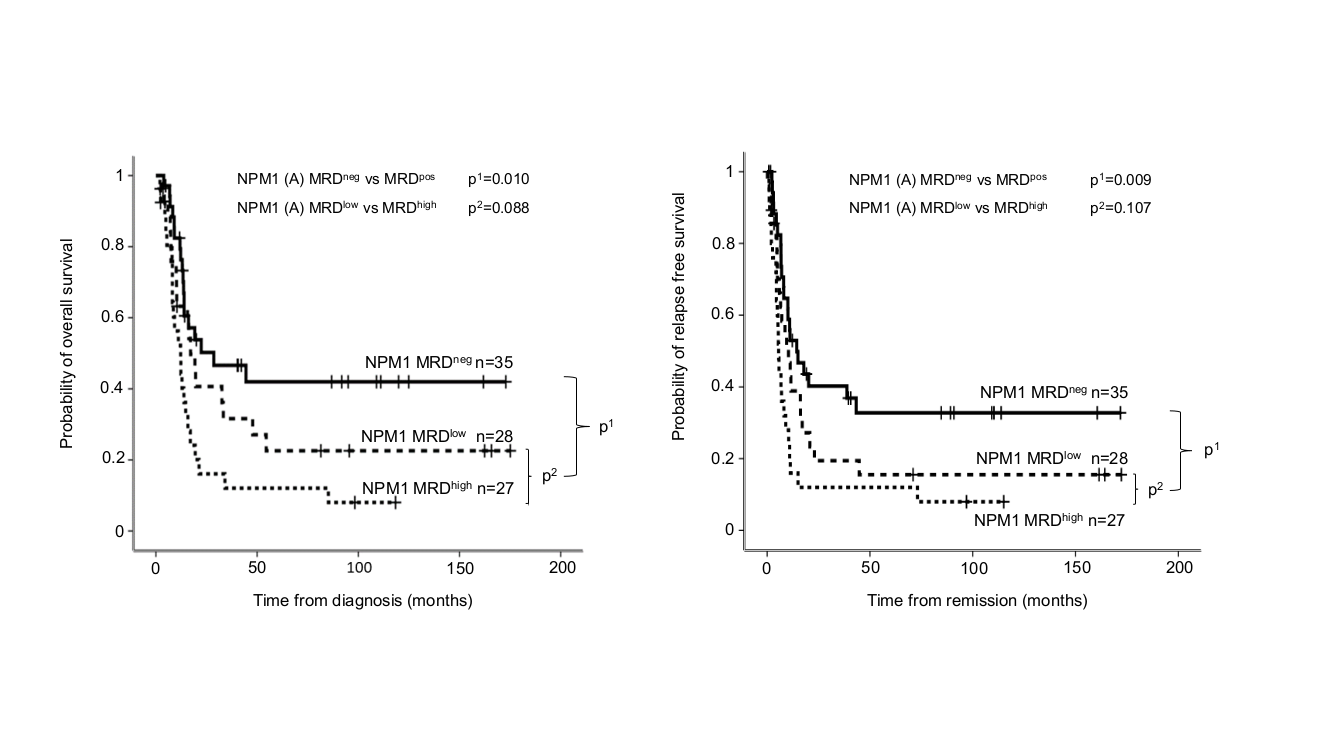

Supplement: S1 Fig — On both panels (A: overall survival; B: relapse free survival), the outcome of NPM1 type-A MRD-negative (MRDneg VAF<0.01%) and MRD-positive (MRDpos VAF>0.01%) subgroups are shown with the associated p1 value. The NPM1 type-A MRD-positive subgroup was further divided in MRD low-positive (MRDlow VAF = 0.01–0.2%) and MRD high-positive (MRDhigh VAF> 0.2%) subgroups, and compared with p2 values. (TIF) [file pone.0253386.s007.tif]

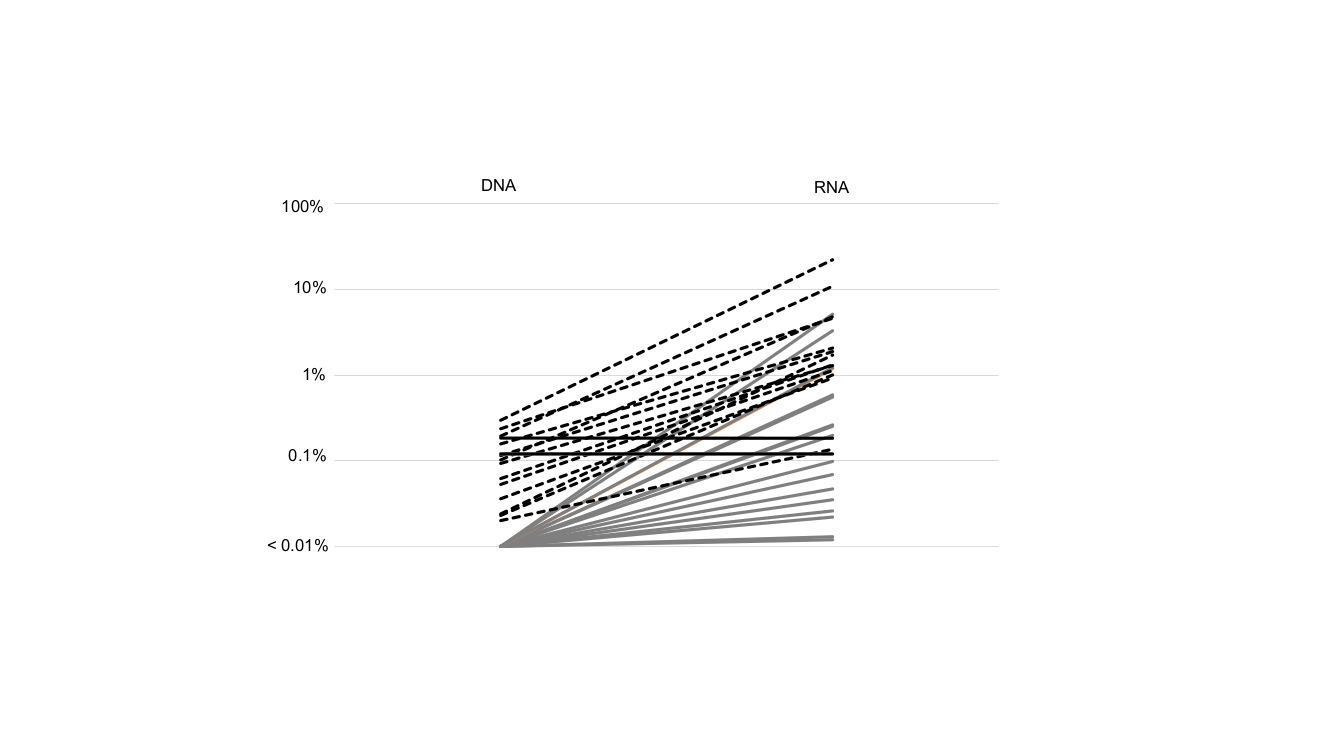

Supplement: S2 Fig — DNA based method describes the variant allele frequencies of mutant NPM1 (NMP1mut/GAPDH ratio), while RNA method showed the NPM1 RNA mutation expression (NPM1mut/ABL1). RNA samples that displayed NPM1mut expression and VAF negativity in the are marked with the grey continuous lines (18 samples, 46%). Samples with at least 0.5 log higher RNA expression level with detectable mutant NPM1 allele frequency on DNA level are shown with black dashed lines (19 samples, 49%). Only two samples (black continuous lines, 5%) showed equivalent NPM1mut RNA expression and DNA allele burden. (TIF) [file pone.0253386.s008.tif]
